# The effect of a supportive program on coping strategies and stress in women diagnosed with breast cancer: A randomized controlled clinical trial

**DOI:** 10.1002/nop2.728

**Published:** 2020-11-30

**Authors:** Elahe Samami, Forouzan Elyasi, Seyed Nouraddin Mousavinasab, Leila Shojaee, Ehsan Zaboli, Zohreh Shahhosseini

**Affiliations:** ^1^ Student Research Committee, Mazandaran University of Medical Sciences Sari Iran; ^2^ School of Medicine Sexual and Reproductive Health Research Center, Psychiatry and Behavioral Sciences Research Center, Addiction Institute Mazandaran University of Medical Sciences Sari Iran; ^3^ School of Health Health Sciences Research Center Mazandaran University of Medical Sciences Sari Iran; ^4^ Department of Surgery School of Medicine Gastrointestinal Cancer Research Center Mazandaran University of Medical Sciences Sari Iran; ^5^ Department of Hematology & Oncology School of Medicine Gastrointestinal Cancer Research Center Mazandaran University of Medical Sciences Sari Iran; ^6^ Sexual and Reproductive Health Research Center Mazandaran University of Medical Sciences Sari Iran

**Keywords:** breast cancer, coping strategies, nurses, nursing, supportive program

## Abstract

**Aim:**

To investigate the effect of a supportive program on coping strategies and stress in women with breast cancer.

**Design:**

A randomized, two‐armed, controlled trial.

**Methods:**

Sixty women were randomly allocated to intervention group (*N* = 30) and control group (*N* = 30). The interventions were held in six sessions, weekly from August 2018–March 2019 It was consisting of education regarding breast cancer; progressive muscle relaxation; stress management; emotional coping; and problem‐solving strategies.

**Results:**

At baseline, there was no difference between the two groups regarding the mean score of coping strategies and stress. Supportive program group participants experienced a significantly higher increase on their problem‐oriented coping strategies score in comparison with the control group. At the same time, scores in emotion‐oriented coping strategies and stress decreased significantly in the intervention group compared with the control group. Result of this study can be used to develop relevant interventions targeting coping strategies to reduce stress among women with breast cancer.

## 1 INTRODUCTION

Breast cancer is the most common cancer in women (Richardson et al., 2017; Yu et al., 2014), accounting for 33% of all types of cancer and 19% of deaths caused by cancer in women (McFarland et al., 2018; Samami et al., 2019, 2020). Breast cancer diagnosis and treatment are one of the main sources of stress and crisis in women's lives (Andolhe et al., 2009; Kim et al., 2010; Samami et al., 2020). Women with breast cancer undergoing chemotherapy, experience a more significant stress level (Samami et al., 2019). These stresses are due to the lack of information, reduced self‐efficacy, inability to the adaptation to their new status, changes in the body image and fear of death (Anusasananun et al., 2013; Leal et al., 2015; Sajadian et al., 2017; Samami et al., 2019; Silva et al., 2017). If these stressors are not properly managed, they can lead to relapses of cancer, prolonged treatment, severe depression, anxiety and reduced quality of life in women diagnosed with breast cancer (Stagl et al., 2015; Walshe et al., 2017).

There are different mechanisms for stress management in these women such as cognitive‐ behavioural therapy (Behzadipoor et al., 2013; Kim et al., 2018; Pelekasis et al., 2016; Sheikh Abumasoudi et al., 2015; Stagl et al., 2015), yoga (Jong et al., 2018; Raghavendra et al., 2009), mindfulness (Carlson et al., 2013; Wurtzen et al., 2015) and hypnosis (Montgomery et al., 2017). Coping strategies are one of the most effective mechanisms in helping breast cancer patients, as these patients need help adapting to their disease effectively and engage with it appropriately (Andolhe et al., 2009; Farajzadegan et al., 2015; Shoaa Kazemi et al., 2014).

Coping includes using the adaptive and maladaptive coping mechanism to control and manage stressful situations and adapt to a threat (Badrian et al., 2014). In fact, coping is an important modifier in the relationship between stress and its side effects (Andolhe et al., 2009; Shoaa Kazemi et al., 2014). More effective, efficient and adaptive coping strategies lead to more decrease in stress (Behzadipoor et al., 2013). The coping strategies are divided into two general categories: Problem‐Focused Coping (PFC) and Emotion‐Focused Coping (EFC) strategies (Folkman & Lazarus, 1988; Folkman et al., 1986). In PFC, all efforts are done to change the leading causes of stress as well as problem‐solving (Ahadi et al., 2014; Andolhe et al., 2009), while, in EFC, efforts are towards regulating the emotional responses to stressful events and emotional reactions (Andolhe et al., 2009; Hajian et al., 2017; You et al., 2018). These strategies do not solve the problem and just relax the patient and reduce the amount of stress (Andolhe et al., 2009). If women diagnosed with breast cancer believe in the controlling effects of stress and its side effects, they may use adaptive coping strategies, but, if this stressful situation is uncontrollable, they will focus on maladaptive strategies (Ahadi et al., 2014). The studies indicate that using adaptive coping strategies are effective in reducing stress, provides social and mental adjustment and enhances the quality of life in women with breast cancer (Behzadipoor et al., 2013; Shoaa Kazemi et al., 2013).

Supportive program is one of the effective mechanisms for improving coping strategies. This program includes providing information and counselling as well as training in coping strategies (Andolhe et al., 2009; Han et al., 2008; Khalili et al., 2013). Women diagnosed with breast cancer that are undergoing chemotherapy, experience more psychological trauma and need interventions to cope with these problems (Nikbakhsh et al., 2014; Pelekasis et al., 2016).

## 2 BACKGROUND

A review of the existing literature shows the controversial results about the effectiveness of psychological interventions to improve coping strategies in women with breast cancer. In one way, it has been showed that cognitive‐behavioural intervention improves PFC, decreases EFC; stress and negative mood in women diagnosed with breast cancer (Behzadipoor et al., 2013; Shoaa Kazemi et al., 2013). Other studies found that cognitive‐behavioural therapy based on positive self‐talk as well as spiritual‐religious intervention lead to enhanced coping strategies in women diagnosed with breast cancer, but the results were not significant (Ghahari et al., 2017; Hamilton et al., 2011). It seems these interventions help women diagnosed with breast cancer to make suitable decisions, improve their self‐care, adapt to their new status and reduce stress by enhancing their information; abilities and self‐confidence (Behzadipoor et al., 2013; Hamzehgardeshi et al., 2017; Heravikarimvi et al., 2006). It should be mentioned that while the results of studies are different, all of these studies emphasize and encounter the need for more studies in regards of coping strategies interventions, in form of supportive and emotional interventions (Ghahari et al., 2017; Hajian et al., 2017).

Women with breast cancer often experience a lack of supportive care and encounter program many psychological traumas that in fact needs professional care. Usually, in countries with low or middle income, supportive care and long‐term follow‐up programs are very limited (Cardoso et al., 2013). In these cases, supportive care programs are not considered as an integral part of the treatment and are often subjected to many restrictions. However, supportive care turned into a structured, mapped out program can be of significant value to all healthcare professionals. Therefore, considering the increasing numbers of breast cancer in low‐ and middle‐income countries, (Ganz et al., 2013; Yip & Taib, 2012) and the crucial effect of supportive care in long‐term treatment of patients, this study was designed.

### 2.1 Aim

This study aims to answer the following question:

1. Does supportive programs compared with routine care improve coping strategies in women with breast cancer?
2. Does supportive programs compared with routine care decrease stress in women with breast cancer?

## 3 METHODS

### 3.1 Design

This is a randomized two‐armed controlled clinical trial, pre‐test posttest and follow‐up for one month after the intervention to determine the effect of a supportive program on coping strategies and stress in women diagnosed with breast cancer.

### 3.2 Setting and sample size

Recruitment took place in the chemotherapy ward of a referral hospital of Sari a city located north of Iran, from August 2018–March 2019. In the power analysis, at least 60 participants (30 in each group) were required for the study with a power of 0.84, to obtain a moderate effect size of 0.5 on EFC score (Shoaa Kazemi et al., 2014) at 5% significance level, a confidence interval of 0.95 and a 20% attrition rate between baseline and follow‐up.

### 3.3 Inclusion criteria

Inclusion criteria were at least elementary level education, stage of I–III breast cancer (according to the patient's medical record), maximum one year after cancer's initial diagnosis, patients undergoing chemotherapy (at least one chemotherapy session), breast cancer with no concurrent history of another cancer, no hospitalization in the psychiatric ward, no using of psychiatric drugs (antipsychotic and antidepressant), no psychiatric disorders (according to each individual's history) and no severe depression and anxiety (according to DASS‐21 questionnaire).

### 3.4 Exclusion criteria

Exclusion criteria consisted of participation in other psychotherapy interventions after inclusion, severe psychiatric symptoms which needed treatment, exacerbation of cancer (incidence of new metastases or development of the disease stages) according to the patient's medical record, participation in educational and counselling courses on breast cancer over the past six months, use of alternative interventions such as traditional medicine and adverse life events, including death of family members (spouse, child, father, mother, sister and brother), divorce, spouse abandonment and severe accident occurred during the study or the past 6 months.

### 3.5 Randomization

In this study, the participants were randomly divided into two groups. The permuted block randomization by computer software was used to select 10 blocks, each with six participants, such that each block had the same number of participants in the intervention and control groups. Sixty envelopes were prepared, and the intervention and control groups were placed in groups I (intervention) or group C (control) in each envelope. The first eligible patient took the first envelope and if group I was written on the envelope, it was included in the intervention group and if group C was written on the envelope, it was included in the intervention group. Whenever, the number of intervention groups reached 10, the first intervention session began for that group. Thus, the intervention group was divided into three groups of 10 eligible participants.

### 3.6 Intervention

A supportive program protocol was developed by extensive review of the existing literature. Then, content validity of the protocol was confirmed by three psychiatrists and one PhD in clinical psychology. The intervention was held in six weekly sessions (one session per week for 90 min in each group). Intervention sessions were held by a Master of Science student in midwifery counselling, whom had completed a 60‐hr life skills workshop and was performed under supervision of second author (F.E, Psychiatrist) and correspondent author (Z.Sh, Ph.D in Reproductive Health). The content of the sessions included education about the following topics: Breast cancer, Diaphragmatic breathing and progressive muscle relaxation technique, Cancer‐related stressors, Stress management, Problem and emotion‐focused coping strategies, Activation strategies, Social support and Spiritual coping strategies. Also, at the end of each session, participants were given a homework assignment to present their feedback in the next session. A summary of protocol content is presented in Table 1. Both intervention and control groups received routine care which included trainings by a chemotherapy nurse or oncologist about postchemotherapy physical and nutritional problems.

**TABLE 1:** A summary of the supportive program sessions' content

Session 1	Introducing the aim of the study, determining participants’ roles, introducing the titles that were presented in the intervention sessions Providing information about breast cancer, its consequences on patients’ lives and treatment Explaining and exercising the diaphragmatic breathing technique Explaining and exercising the progressive muscle relaxation technique Homework assignment (exercising the diaphragmatic breathing and the progressive muscle relaxation technique)
Session 2	Reviewing the content of the last session Assessing and discussing homework assignments Explaining the stressors associated with cancer Providing information about signs and sources of stress Stress management strategies Homework assignment (exercising the progressive muscle relaxation technique, reviewing the stress related to breast cancer and discussing it)
Session 3	Reviewing the content of the last session Assessing and discussing homework assignments Providing explanations and counselling about coping and emotion‐focused coping strategies Providing information and counselling on emotion expression strategies Homework assignment (practicing the writing techniques in expressing excitement at home)
Session 4	Reviewing the content of the last session Assessing and discussing homework assignments Providing information about coping and problem‐focused coping strategies Providing information, counselling and practicing on problem‐solving strategies Homework assignment (practicing the problem‐solving techniques on the recent problem and record its process)
Session 5	Reviewing the content of the last session Assessing and discussing homework assignments Providing information about activation strategies Homework assignment (practicing cognitive distortion control strategies and activation strategies at home)
Session 6	Reviewing the content of the last session Assessing and discussing homework assignments Providing information about social support Providing information about spiritual coping strategies Summarizing what has been learned and emphasize continuing to practicing the techniques and strategies presented in previous sessions

### 3.7 Outcomes

The primary outcomes consisted of coping strategies and included PFC and EFC. These continuous variables were measured by Ways of Coping Questionnaire (WOCQ). The secondary outcome included stress. This continuous variable was evaluated by Depression‐Anxiety‐Stress Scale (DASS‐21).

### 3.8 Measurements

#### 3.8.1 Socio‐demographic‐clinical characteristics

This questionnaire consisted of questions about women's age, their spouse's age, number of children, level of education, marital status, employment status, duration of cancer, number of chemotherapy sessions, surgery type, stage of cancer, depression score, anxiety score and social support score.

#### 3.8.2 Ways of Coping Questionnaire

This questionnaire was provided by Lazarus and Folkman in 1980 and was revised in 1985. This questionnaire has been divided into PFC (with four subscales: Seeking social support [six items], Accepting responsibility [four items], Planful problem solving [six items], Positive reappraisal [seven items]) and EFC (with four subscales: Confronting coping [six items], Distancing [six items], Escape‐avoidance [eight items] and Self‐controlling [seven items]). Scoring was based on Likert scale 0–3 and because the number of PFC and EFC items varies, their scores as well as the scores of their subscales were calculated and reported from 100 to be comparable (Folkman & Lazarus, 1988; Folkman et al., 1986). Ahadi et al. reported that the validity of this questionnaire in Iranian context on women diagnosed with breast cancer and healthy samples were 0.39–0.65 and 0.57–0.8, respectively. Also, the Cronbach's alphas were 0.66 and 0.87 in above‐mentioned groups, as well (Ahadi et al., 2014). This questionnaire was completed at baseline, immediately after completing the intervention and one month after the intervention in the intervention group. In the control group, participants completed it at baseline, 6 weeks and one month after completing the first questionnaire.

#### 3.8.3 Depression‐Anxiety‐Stress Scale

Depression‐Anxiety‐Stress Scale questionnaire was introduced by Lovibond and Lovibond in 1995 (Lovibond & Lovibond, 1995). This instrument is a set of three self‐report scales designed to measure the emotional states of depression, anxiety and stress. Scoring was based on Likert scale (0–3) and the range of total score was 0–42. As the current scale is the short form of the main one (42 items), the total score of each subscale needed to be multiplied by two. Antony et al. reported a 0.94, 0.87 and 0.91 for Cronbach's alpha for depression, anxiety and stress, respectively (Antony et al., 1998; Asghari et al., 2008; Sahebi et al., 2005). This questionnaire was completed by all participants in both groups at the beginning of the present study.

#### 3.8.4 Medical Outcomes Study‐Social Support Scale

To assess social support as a main covariate in coping strategies, the Medical Outcomes Study‐Social Support Scale was completed by participants. This scale was provided by Sherbourne and Stewart in 1991 to assess the extent of which a person has the support of others in facing stressful life events (Sherbourne & Stewart, 1991). It has 19 questions and five subscales including: tangible support, emotional support, information, kindness and positive social interaction. Scoring was based on Likert scale (1–5), and the range of total scores was 19–95. The reliabilities of this questionnaire were approved by the Cronbach's alpha for emotional support, informational support, tangible support, positive social interaction and kindness at 0.96, 0.92, 0.94, 0.91 and 0.97, respectively (Barzegar et al., 2017; Schmidt et al., 2012; Sherbourne & Stewart, 1991).This questionnaire was completed by all participants in both groups at the beginning of the present study.

### 3.9 Data analysis

Data were entered into SPSS −20. Data description was performed with the measures of the descriptive statistics such as mean and standard deviation for continuous variables such as duration of cancer diagnosis and number of chemotherapy sessions and relative frequency for categorical variables such as surgery type and stage of cancer. If the Shapiro–Wilk test failed to show the normal distribution of the variables, the non‐parametric tests were used. Furthermore, independent *t* test, paired *t* test, chi‐square and Fisher exact tests were conducted to compare the participants' socio‐demographic and clinical characteristics. Comparison between groups on PFC, EFC and their subscale's mean scores were performed by Mann–Whitney *U* test, as for the stress score the independent *t* test was conducted. As data were being collected from the same participants over time, observations were correlated and hence, an analytical method such as generalized estimating equations was needed to consider such correlations. So, for determining the effects of time and intervention on PFC, EFC and stress score this test was employed. In this study, effect size (Cohen's *d*) were calculated and the level of significance in this study was set at <0.05.

## 4 RESULTS

**FIGURE 1:**
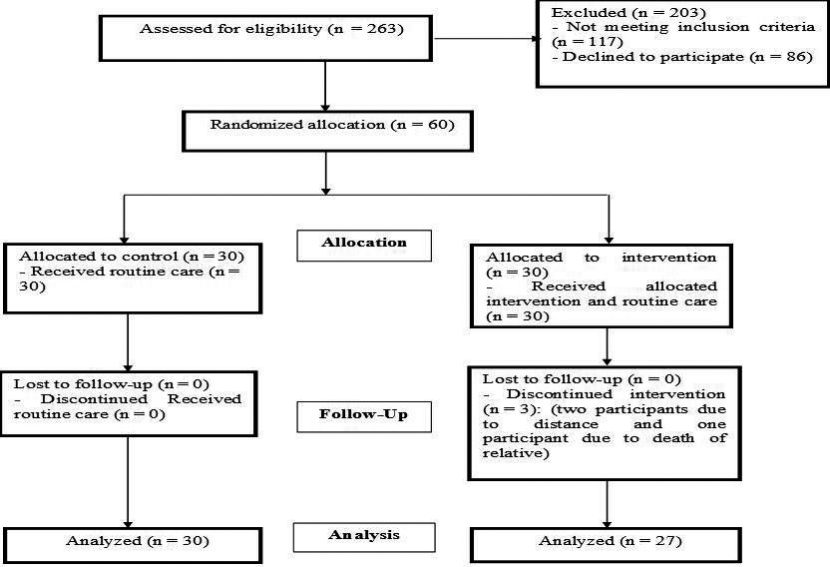
CONSORT 2010 flow diagram

Three participants withdrew from the intervention group in this study and data retrieved from 57 participants (27 participants in intervention group and 30 participants in control group) were finally analysed (Figure 1). There was no significant difference between the two groups in socio‐demographic characteristics at the baseline (Table 2). The Mann–Whitney *U* test showed no significant differences between the intervention (45.19 *SD* 13.85) and control groups (47.10 *SD* 15.09), regarding the PFC' score at the baseline (*p* = .623). This score increased in intervention group (73.69 ± 7.62) compared with control group (46.57 *SD* 11.70), immediately after completing the intervention (*p* < .001, very large effect size = 2.75) and one month after the intervention (72.73 *SD* 7.56 vs. 46.57 *SD* 12.63, *p* < .001, very large effect size = 2.48; Table 3). As shown in Table 4, no significant differences were found between intervention and control groups at the baseline in subscales scores of the PFC such as seeking social support (*p* = .356), accepting responsibility (*p* = .941), Planful (planned) problem‐solving (*p* = .180) and positive reappraisal (*p* = .388). These subscale scores were significantly increased in intervention group compared with control group immediately after completing the intervention (*p* < .001) and one month after the intervention (*p* < .001). The effect size of the subscales indicated a very large effect size that indicates the magnitude of the intervention effect.

**TABLE 2:** Participants' socio‐demographic and clinical characteristics

Variables	Groups		*p*‐value
	Intervention (*n* = 27)	Control (*n* = 30)	
Age (year) (*M* ± *SD*)	43.81 (7.41)	44.00 (7.27)	.925
Number of children (*M* ± *SD*)	2.15 (0.86)	2.10 (0.75)	.824
Duration of cancer diagnosis (month; *M* ± *SD*)	4.59 (2)	5.40 (1.49)	.089
Number of chemotherapy sessions (*M* ± *SD*)	3.81 (1.71)	3.77 (1.19)	.902
Depression score (*M* ± *SD*)	6.74/21.00 (4.61)	6.93/21.00 (4.19)	.891
Anxiety score (*M* ± *SD*)	4.88/21.00 (3.56)	4.40/21.00 (3.76)	.520
Social support score (*M* ± *SD*)	76.74/95.00 (5.44)	76.26/95.00 (5.89)	.755
Level of education *N* (%)			
High school and lower	20 (74.07)	24 (80)	.847
Upper from high school	7 (25.93)	6 (20)	
Upper from high school	6 (22.22)	8 (26.67)	
Marital status *N* (%)			
Married	26 (96.30)	29 (96.67)	1
Single	1 (3.70)	1 (3.33)	
Employment status *N* (%)			
Housewife	25 (92.60)	26 (86.67)	.673
Employed	2 (7.40)	4 (13.33)	
Type of surgery *N* (%)			
Unilateral mastectomy	7 (25.93)	7 (23.33)	1
Bilateral mastectomy	0 (0)	0 (0)	
Lumpectomy	17 (62.96)	20 (66.67)	
No surgery	3 (11.11)	3 (10)	
Stage of cancer *N* (%)			
I	8 (29.63)	7 (23.33)	.764
II, III	19 (70.37)	23 (23.23)	

**TABLE 3:** Mean score, standard deviation, effect size of coping and stress' scores, at the baseline, immediately and one month after the intervention^a^

Outcomes	Time	Groups			*p*‐Value
		Intervention (mean ± *SD*)	Control (mean ± *SD*)	Effect size (AMD)^b^	
Problem‐focused coping' score (total score ranged between 0–100)	Baseline	45.19 (13.85)	47.10 (15.09)	—	.623
	Immediately after the intervention	73.69 (7.62)	46.57 (11.70)	2.75	<.001
	One month after the intervention	72.73 (7.56)	46.57 (12.63)	2.48	<.001
Emotion‐focused coping' score (total score ranged between 0–100)	Baseline	54.13 (8.19)	51.68 (9.31)	—	.299
	Immediately after the intervention	41.92 (6.42)	55.14 (8.44)	1.70	<.001
	One month after the intervention	40.32 (6.34)	53.08 (8.60)	1.67	<.001
Stress score (total score ranged between 0–21)	Baseline	17.62 (4.93)	16.01 (5.09)	—	.226
	Immediately after the intervention	8.59 (3.17)	15.86 (4.19)	1.94	<.001
	One month after the intervention	8.22 (2.95)	17.26 (4.28)	2.43	<.001

^a^ The results of the Mann–Whitney *U* test.

^b^ Adjusted mean difference.

**TABLE 4:** Mean score (out of 100), standard deviation and effect size of subscales of coping' scores at the baseline, immediately and one month after the intervention^a^

Outcomes	Time	Groups			*p*‐Value
		Intervention (mean ± *SD*)	Control (mean ± *SD*)	Effect size (AMD)^b^	
Problem‐focused coping					
Seeking social support	Baseline	51.44 (12.94)	53.33 (14.99)	—	.356
	Immediately after the intervention	81.48 (6.89)	54.25 (11.64)	2.81	<.001
	One month after the intervention	81.48 (5.96)	53.33 (12.43)	2.83	<.001
Accepting responsibility	Baseline	45.98 (15.39)	46.38 (18.39)	—	.941
	Immediately after the intervention	69.75 (12.89)	45.01 (11.07)	2.06	<.001
	One month after the intervention	69.75 (12.47)	45.27 (13.61)	1.87	<.001
Planful problem‐solving	Baseline	39.91 (16.01)	42.77 (15.51)	—	.180
	Immediately after the intervention	69.75 (20.63)	42.59 (13.39)	1.58	<.001
	One month after the intervention	65.43 (9.15)	42.96 (14.14)	1.86	<.001
Positive reappraisal	Baseline	43.91 (17.28)	45.87 (19.14)	—	.388
	Immediately after the intervention	72.66 (9.84)	44.28 (15.06)	2.21	<.001
	One month after the intervention	73.19 (10.49)	44.60 (15.84)	2.10	<.001
Emotion‐focused coping					
Confrontive coping	Baseline	43.62 (8.67)	44.81 (10.30)	—	.474
	Immediately after the intervention	34.56 (6.77)	47.03 (8.84)	1.57	<.001
	One month after the intervention	31.48 (5.11)	45.18 (8.96)	1.85	<.001
Distancing	Baseline	56.58 (12.70)	54.25 (13.96)	—	.720
	Immediately after the intervention	43.62 (8.81)	63.14 (10.86)	1.96	<.001
	One month after the intervention	41.97 (8.05)	58.70 (11.36)	1.68	<.001
Escape‐avoidance	Baseline	55.86 (14.07)	53.19 (16.58)	—	.328
	Immediately after the intervention	41.51 (9.20)	55.41 (14.76)	1.11	<.001
	One month after the intervention	39.81 (9.41)	55.01 (14.28)	1.24	<.001
Self‐controlling	Baseline	59.08 (8.88)	53.65 (9.51)	—	.007
	Immediately after the intervention	47.26 (9.87)	54.92 (10.21)	0.76	.004
	One month after the intervention	47.08 (8.69)	52.85 (8.87)	0.65	.019

^a^ The results of the Mann–Whitney *U* test.

^b^ Adjusted mean difference.

According to Table 3, there were no significant differences between the mean score of EFC at the baseline in intervention and control groups (54.13 *SD* 8.19, 51.68 *SD* 9.31, *p* = .299). This score significantly decreased in intervention group compared with control group immediately after completing the intervention (41.92 *SD* 6.42 vs. 55.14 *SD* 8.44, *p* < .001, large effect size = 1.70) and one month after intervention. (40.32 *SD* 6.34 vs. 53.08 *SD* 8.60, *p* < .001, large effect size = 1.67). Mean score of EFC subscales, such as confronting coping (*p* = .474), distancing (*p* = .720), escape‐avoidance (*p* = .328), except self‐controlling (*p* = .007), was not significantly different in the two groups at the baseline. As shown in Table 4, these subscale scores were significantly decreased in intervention group compared with control group immediately after completing the intervention (*p* < .001) and one month after the intervention (*p* < .001). The effect size of the subscales varied from a moderate effect size for self‐controlling subscale, a large effect size for escape‐ avoidance and to a very large effect size in the other subscales.

The result of the independent *t* test showed there were no significant differences between mean score of stress in intervention group (17.62 *SD* 4.93) and control group (16 *SD* 5.09) at the baseline (*p* = .226). But, this score decreased in intervention group compared with control group immediately after completing the intervention (8.59 *SD* 3.17 vs. 15.86 *SD* 4.19, *p* < .001, large effect size = 1.94) and one month after the intervention (8.22 *SD* 2.95 vs. 17.26 *SD* 4.28, *p* < .001, large effect size = 2.43).

The generalized estimating equation showed, scores of PFC in intervention group increased to 17.12 compared with control group (*p* < .001).The results of Table 5 show scores of PFC immediately after completing the intervention with the regression coefficient (*β*) increasing to 13.22 and in one month after the intervention increasing to a 12.76 (*p* < .001). Also, the score of EFC in intervention group decreased to 7.84 compared with control group (*p* < .001). The results of this study show, EFC scores immediately after completing the intervention with decrease in regression coefficient (*β*) to a 3.96 and in one month after the intervention with decrease to a 5.80 (*p* < .001). In addition, stress score in intervention group decreased to 4.89 compared with control group (*p* < .001). Also, immediately after completing the intervention, stress score reduced with regression coefficient (*β*) decreasing to 4.35 and decreasing to 5.80 in one month after the intervention (*p* < .001). The results of the generalized estimating equation indicate that the supportive program interventions have a significant effect in regards to score of PFC, EFC and stress, by controlling the effect of time measurements of consequences.

**TABLE 5:** The results of the generalized estimating equations for the effects of supportive program on coping and stress

Outcome	Time	*β*	*SE*	*p*‐Value
Problem‐focused coping	Intervention vs. control group	17.12	2.82	<.001
	Immediately after the intervention	13.22	2.23	<.001
	One month after the intervention	12.76	2.14	<.001
Emotion‐focused coping	Intervention vs. control group	−7.84	1.95	<.001
	Immediately after the intervention	−3.96	1.24	<.001
	One month after the intervention	−5.80	1.19	<.001
Stress	Intervention vs. control group	−4.89	1.01	<.001
	Immediately after the intervention	−4.35	0.67	<.001
	One month after the intervention	−3.78	0.81	<.001

## 5 DISCUSSION

This study aimed to determine the effect of a supportive program on coping strategies and stress in women diagnosed with breast cancer. This program statistically increased the PFC score and decreased EFC and stress scores in the intervention group compared with the control group that is in accordance with the previous studies (Behzadipoor et al., 2013; Kang & Oh, 2012; Loh & Quek, 2011; Shoaa Kazemi et al., 2013). It seems a wide variety of services are required to manage challenges surrounding cancer diagnosis, as well as the physical and emotional side effects of cancer treatment, problem‐solving skills and stress management that can eventually lead to improvement in coping strategies; however, some studies have failed to demonstrate this concept (Cousson‐Gelie et al., 2011; Ghahari et al., 2017; Hamilton et al., 2011). An explanation for this difference might be due to the time of intervention for example the previous studies conducted the intervention at the end of the chemotherapy and radiotherapy sessions (Ghahari et al., 2017; Hamilton et al., 2011) and at this stage patient has already coped with the new circumstances and the supportive program was not effective.

Another reason for the insignificance of the above studies could be a limited number of sessions, a short interval between intervention sessions and the smaller sample size. For example, Hamilton et al. implemented their intervention session in the form of one workshop session for two hours and Cousson‐Gelie et al. held the intervention sessions in eight weeks (two sessions per week) (Cousson‐Gelie et al., 2011; Hamilton et al., 2011). In addition, Ghahari et al. included 45 women with breast cancer (three groups, each with 15 patients; Ghahari et al., 2017). In the present study, 57 patients were enrolled and intervention sessions were held for 90 min within six weeks.

Consistent with the literature (Allen et al., 2002; Carlson et al., 2013; Chen et al., 2018; Kang & Oh, 2012; Pelekasis et al., 2016; Sheikh Abumasoudi et al., 2015), this research found that supportive program by using breathing technique, relaxation and problem‐solving strategies lead to reduction of stress score in women diagnosed with breast cancer. In this study, attempts were made to reduce the stress of participants by identifying sources of stress and practicing the techniques provided. However, the findings of the current study do not support the previous research (Orsak et al., 2015). This result may be explained by the type of intervention performed, the smaller number of intervention sessions (four sessions), the shorter duration (30 min) for each session and the limited sample size (15 women). Another reason for the discrepancy was the lake of attention to psychiatric disorders. In this study, women with history of severe psychiatric disorders, hospitalization in the psychiatric ward, using psychiatric drugs, major depressive disorder and anxiety disorders were excluded from the study.

### 5.1 Limitation

The random allocation of the samples, using the standard questionnaires and intervention based on a protocol designed by the research team could be mentioned as the strengths of the present study. However, the study could not deal with many factors, including the individuals' perception of the disease and individuals' culture that are important in choosing the coping strategies and thus this was one of the limitations of the study. Another limitation of this study was that since patients were referred for chemotherapy on different days, patients from the two groups of intervention and control were more likely to meet and this issue may prone the study to contamination bias. Although this possibility is very small due to random sampling, two separate rooms in the chemotherapy ward the number of different chemotherapy sessions and receiving chemotherapy on different days. Due to non‐masked design of this study at the participants and outcome assessor level, the results of this study might be prone to performance and detection biases.

## 6 CONCLUSION

In conclusion as in developing countries delivery of supportive care for breast cancer women is often a low priority (Cardoso et al., 2013; Ganz et al., 2013), it is important that healthcare professionals be provided with non‐pharmacological interventions such as supportive programs to help breast cancer survivors in encountering with their new status. The results and protocol of this study can be used in practice to improve coping strategies and reduce stress in women with breast cancer. Empowering healthcare professionals with providing these services will offer patients alternative treatment methods.

## CONFLICT OF INTERESTS

The authors have no conflicts of interest relevant to this article.

## ETHICAL APPROVAL

This is a randomized controlled clinical trial, which has been registered in Iran National Committee for Ethics in Biomedical Research (Code: IR.MAZUMS.REC.1397.155) and in Iranian Registry of Clinical Trials (Code: IRCT20150608022229N4). Written informed consents were obtained from all participants and they were ensured that their identity would be kept anonymous throughout the study. Participants, who voluntarily withdrew (in intervention group, *N* = 3) from the study, received routine care services as before.

## Data Availability

Data are available on request due to privacy/ethical restrictions.
